# Supplementary material for: ZnO Synthesized Using Bipolar Electrochemistry: Structure and Activity
Source: Materials (Basel). 2019 Feb 11;12(3):535. doi: 10.3390/ma12030535 (PMC6384836; doi:10.3390/ma12030535)
Supplement: Supplementary file 1 [file materials-12-00535-s001.pdf]

materials

MDPI

Article

ZnO Synthesized Using Bipolar Electrochemistry: Structure and Activity

Arya Hakimian, Steven McWilliams and Anna Ignaszak *

Department of Chemistry, University of New Brunswick, 30 Dineen Drive, Fredericton, NB, E3B 5A3, Canada.
* Correspondence: Anna.Ignaszak@unb.ca; Tel: +86-150-6261-9128

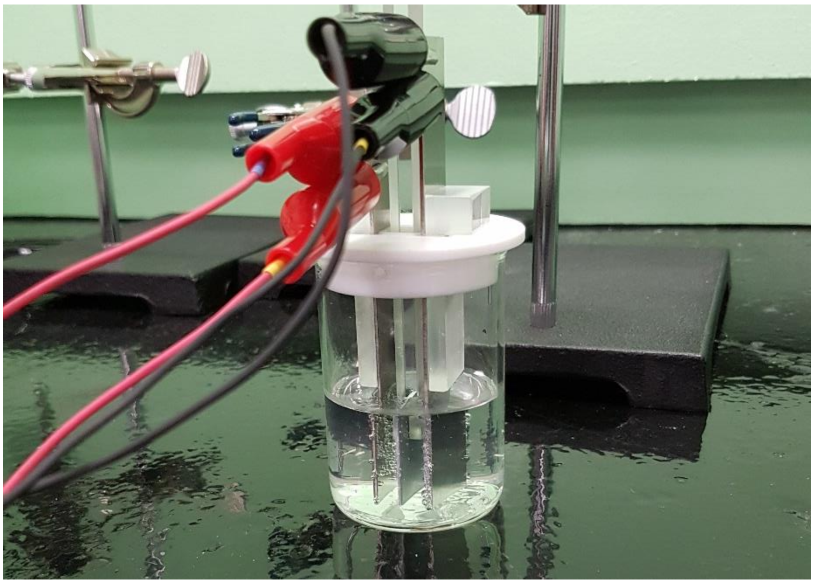

Figure S1. Reaction cell setup used for bipolar electrochemical synthesis.

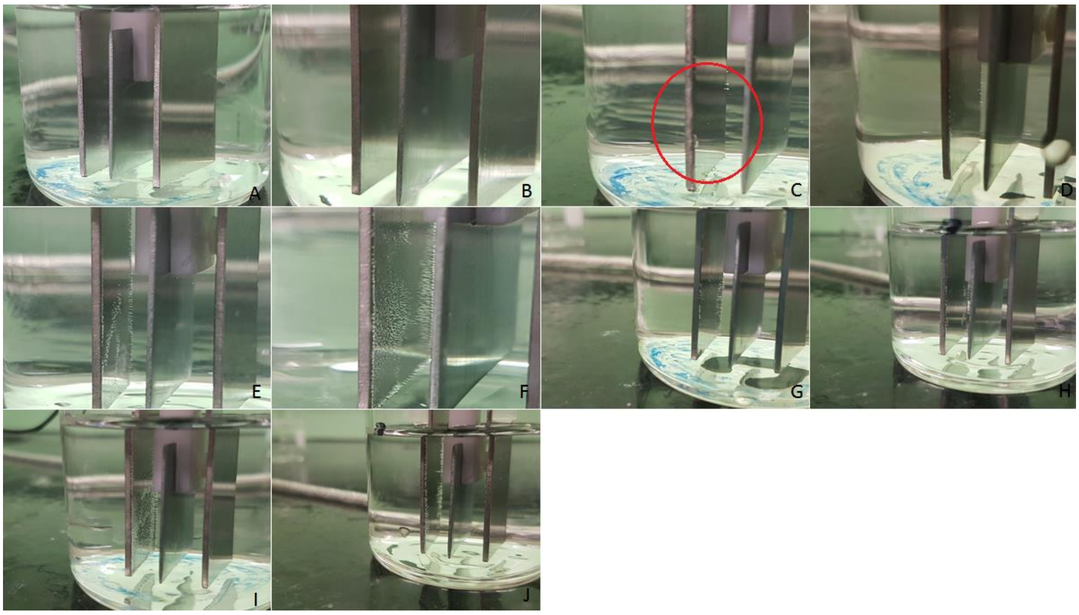

Figure S2. Images of the qualitative varying potential analysis performed at (**A**) 1.32 V, (**B**) 5 V, (**C**) 10 V, (**D**) 20 V, (**E**) 30 V, (**F**) 40 V, (**G**) 50 V, (**H**) 55 V, (**I**) 60 V and (**J**) 62.5 V.

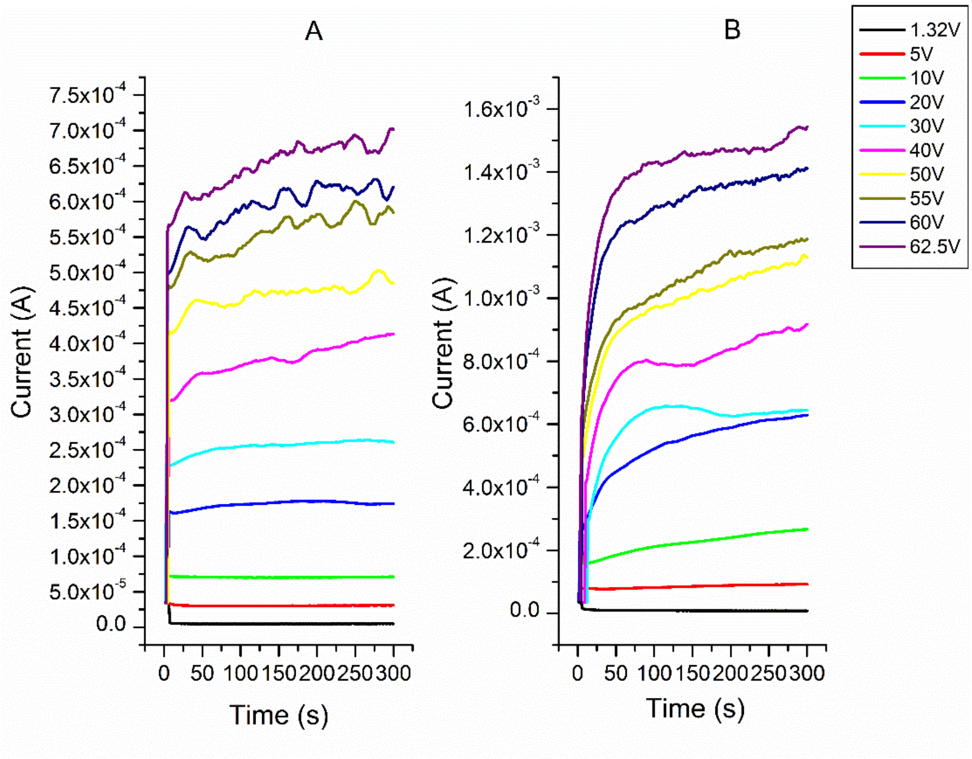

Figure S3. *I*–*t* graphs of current generation at varying applied potentials for both systems (**A**) without and (**B**) with a bipolar electrode (BPE) present.

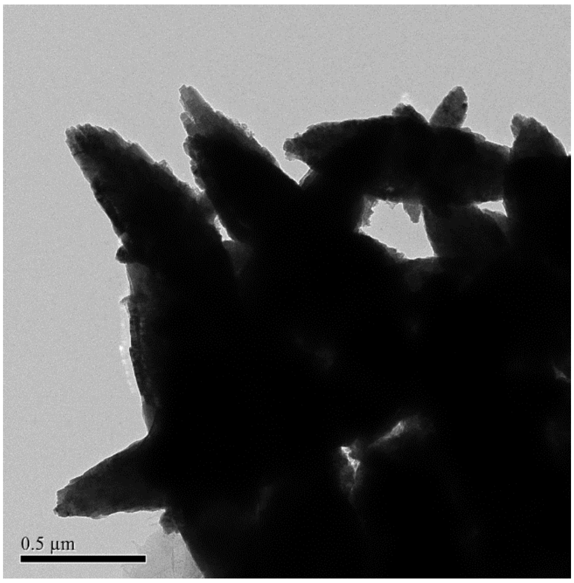

Figure S4. TEM image of a 2-h room temperature trial collected via evaporation showing the presence of larger agglomerates without any indication of smaller nanoparticles.

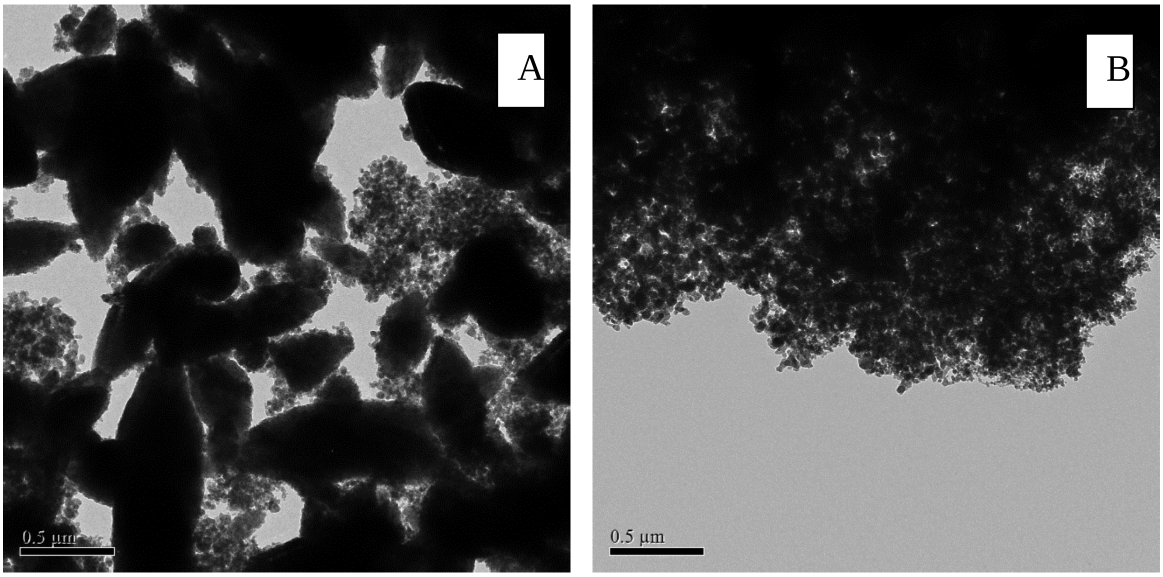

Figure S5. TEM images of 2-h trials collected via filtration performed at (**A**) room temperature and (**B**) in an ice bath (~5°C).

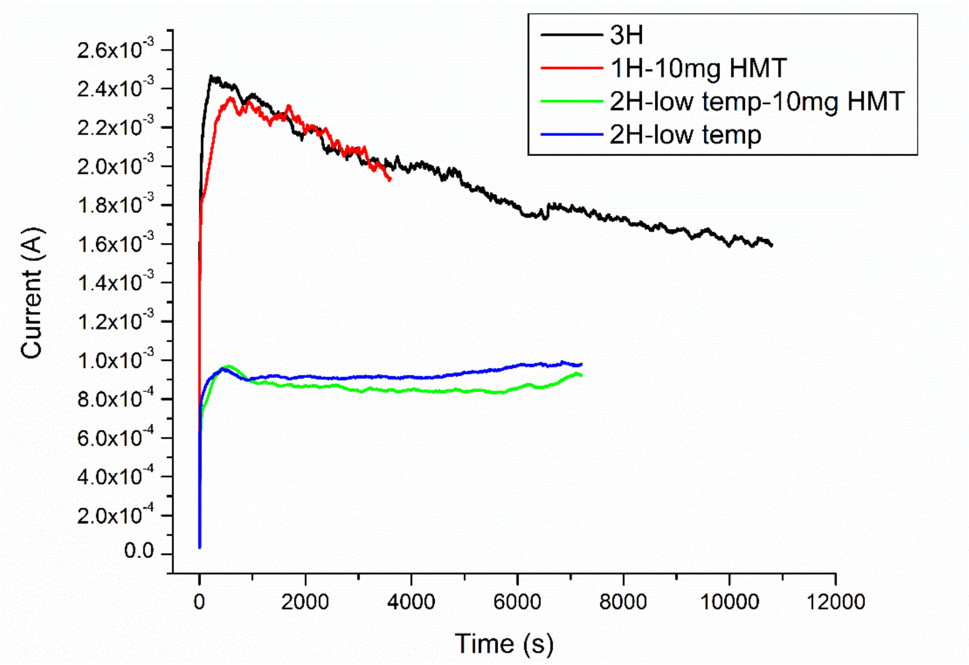

Figure S6. Overlay of the *i–t* graphs generated from trial data, which demonstrates the magnitude of current generated under various conditions.

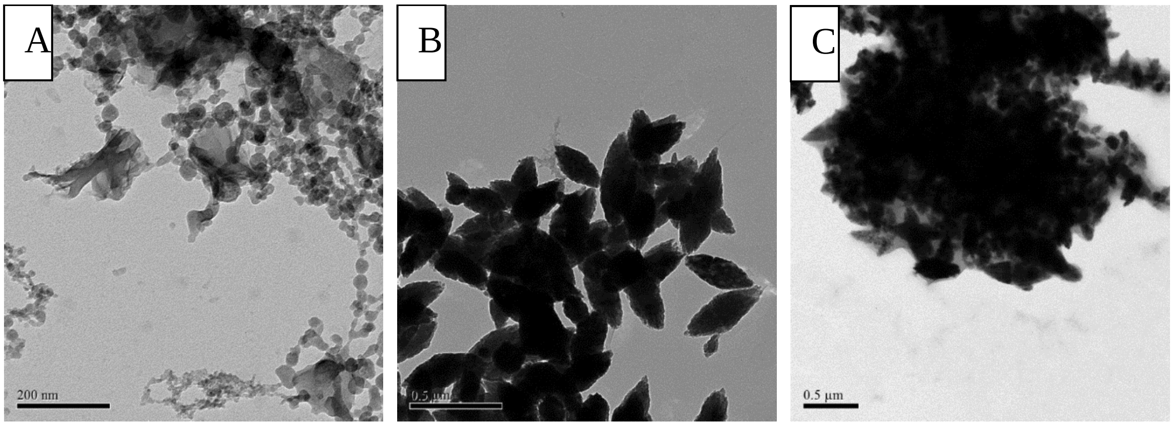

Figure S7. TEM images of 2-h room temperature trial with 10 mg hexamethylenetetramine (HMT) collected via evaporation resulting in both larger agglomerates and smaller nanoparticles (**A,B**) and 2-h room temperature trial with 10 mg PVP resulting solely in larger agglomerates (**C**).

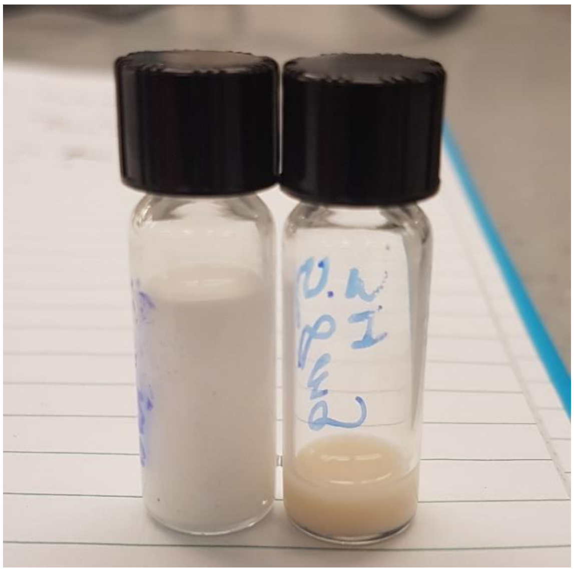

Figure S8. Comparison of pure white commercial ZnO (**left**) and 2-h synthesized sample (**right**) in ethanol depicting the off-white color obtained for the synthesized samples.

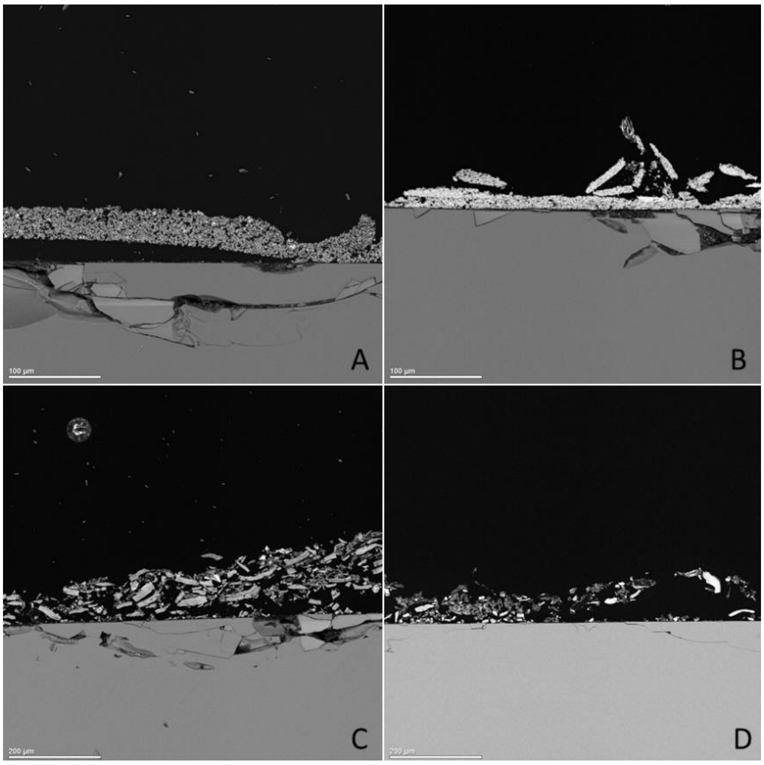

Figure S9. SEM cross-sectional images of Fluorine doped tin oxide (FTO)-coated ZnO electrodes for (**A**) Com, (**B**) 3He, (**C**) 3Hf and (**D**) 2H samples.

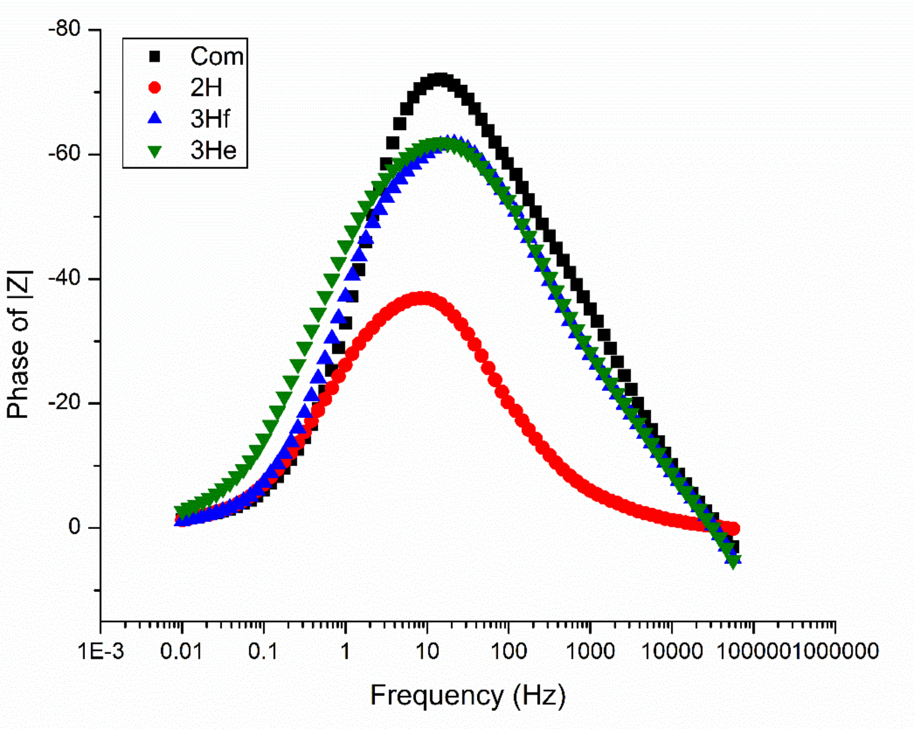

Figure S10. Bode diagram of the illuminated ZnO-based electrodes with the commercial and synthesized samples.

Table S1. Comparison of charger-transfer resistance (*R_{ct}*), transport resistance (*R_{tr}*), an effective diffusion length (*L_n*) and free-electron lifetimes (*t_n*) under illumination estimated by impedance modelling and from Equations (13) and (14) in main manuscript.

ZnO	Dark				
	**L (μm)**	**R_{ct} ($\Omega \times 10^6$)**	**R_{tr} (Ω)**	**$\sqrt{R_{ct}/R_{tr}}$**	**L_n ($\mu m \times 10^3$)**
**Com**	65.5	94	245.0	619.6	40.5
**2H**	84.3	1.6	112.2	119.4	10.1
**3He**	54.4	1.2	82.5	119.0	6.5
**3Hf**	80.2	4.5	130.8	185.6	14.9

ZnO	Illuminated					
	**L (μm)**	**R_{ct} (Ω)**	**R_{tr} (Ω)**	**$\sqrt{R_{ct}/R_{tr}}$**	**L_n ($\mu m \times 10^2$)**	**τ_n (s)**
**Com**	65.5	4558.5	245.0	4.2	2.7	0.27
**2H**	84.3	994.6	112.2	2.9	2.4	0.21
**3He**	54.4	4104.4	82.5	7.0	3.8	0.25
**3Hf**	80.2	1947.7	130.8	3.8	3.0	0.35
